# Device‐Algorithm Co‐Optimization for an On‐Chip Trainable Capacitor‐Based Synaptic Device with IGZO TFT and Retention‐Centric Tiki‐Taka Algorithm

**DOI:** 10.1002/advs.202303018

**Published:** 2023-08-09

**Authors:** Jongun Won, Jaehyeon Kang, Sangjun Hong, Narae Han, Minseung Kang, Yeaji Park, Youngchae Roh, Hyeong Jun Seo, Changhoon Joe, Ung Cho, Minil Kang, Minseong Um, Kwang‐Hee Lee, Jee‐Eun Yang, Moonil Jung, Hyung‐Min Lee, Saeroonter Oh, Sangwook Kim, Sangbum Kim

**Affiliations:** ^1^ Department of Materials Science & Engineering Inter‐university Semiconductor Research Center Research Institute of Advanced Materials Seoul National University Seoul 08826 Republic of Korea; ^2^ Device Solutions Samsung Electronics Pyeongtaek 17786 Republic of Korea; ^3^ Department of Semiconductor System Engineering Korea University Seoul 02841 Republic of Korea; ^4^ School of Electrical Engineering Korea University Seoul 02841 Republic of Korea; ^5^ Samsung Advanced Institute of Technology (SAIT) Samsung Electronics Suwon‐si 16678 Republic of Korea; ^6^ Department of Electrical and Electronic Engineering Hanyang University Ansan 15588 Republic of Korea

**Keywords:** device‐algorithm co‐optimization, indium gallium zinc oxide thin film transistor (IGZO TFT), in‐memory computing, neuromorphic, tiki‐taka algorithm

## Abstract

Analog in‐memory computing synaptic devices are widely studied for efficient implementation of deep learning. However, synaptic devices based on resistive memory have difficulties implementing on‐chip training due to the lack of means to control the amount of resistance change and large device variations. To overcome these shortcomings, silicon complementary metal‐oxide semiconductor (Si‐CMOS) and capacitor‐based charge storage synapses are proposed, but it is difficult to obtain sufficient retention time due to Si‐CMOS leakage currents, resulting in a deterioration of training accuracy. Here, a novel 6T1C synaptic device using only n‐type indium gaIlium zinc oxide thin film transistor (IGZO TFT) with low leakage current and a capacitor is proposed, allowing not only linear and symmetric weight update but also sufficient retention time and parallel on‐chip training operations. In addition, an efficient and realistic training algorithm to compensate for any remaining device non‐idealities such as drifting references and long‐term retention loss is proposed, demonstrating the importance of device‐algorithm co‐optimization.

## Graphical abstract

A novel 6T1C synaptic device based on indium gallium zinc oxide thin film transistor (IGZO TFT) and capacitor and a novel optimized training algorithm, retention‐centric Tiki‐Taka algorithm, is proposed. Through a new training scheme by co‐optimizing the device and algorithm, modified national institute of standards and technology (MNIST) on‐chip training accuracy of over ≈97% even in wide retention requirements is obtained.

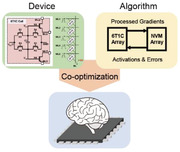

## 1 Introduction

The Analog in Memory Computing (AiMC) system has the advantage of enabling low‐power operation compared to conventional computing systems due to parallelization in the operation of neurons and synapses.^[^ ^1^ ^]^ Because a key element in the AiMC system is analog‐based system devices,^[^ ^2^ ^]^ several resistive switching devices including phase change memory,^[^ ^3^ ^]^ ferroelectric device,^[^ ^4^, ^5^, ^6^, ^7^ ^]^ filamentary resistive random access memory (RRAM),^[^ ^8^, ^9^, ^10^ ^]^ non‐filamentary RRAM,^[^ ^11^ ^]^ spintronics device^[^ ^12^, ^13^ ^]^ have been used to implement the synaptic element. However, resistive switching device has disadvantages in that the weight update linearity and symmetry are not sufficient, and the mechanism of conductance modulation is typically a random process in an atomic‐level change based on electro‐dynamics,^[^ ^14^ ^]^ making it difficult to precisely control its resistance and achieve excellent device variations.^[^ ^15^ ^]^

Recently, as a candidate for the synaptic device, charge storage synapses based on silicon complementary metal‐oxide semiconductor (Si‐CMOS) and capacitors have been proposed, and it has shown the highest level of weight update linearity and symmetry.^[^ ^16^, ^17^, ^18^ ^]^ However, it has a retention problem in that the charge stored in the capacitor is rapidly leaked through the Si transistor. In training the modified national institute of standards and technology (MNIST) dataset and convolutional neural network (CNN), the test error according to the retention time has also been shown.^[^ ^16^ ^]^ To solve the retention problem that occurs in capacitor‐based synapses, a synaptic device using an indium gallium zinc oxide thin film transistor (IGZO TFT) with low leakage current^[^ ^19^, ^20^, ^21^, ^22^ ^]^ and a capacitor have also been devised.^[^ ^23^ ^]^ However, because the IGZO TFT cannot fabricate p‐channel metal‐oxide semiconductor (PMOS) that charge a fixed current, there are research cases that only conducted inference,^[^ ^23^ ^]^ and no synaptic device capable of on‐chip training has been devised.^[^ ^24^ ^]^

Here, we report an IGZO TFT and capacitor‐based synaptic device capable of linear and symmetric weight updates for on‐chip training. We devised a novel 6T1C synaptic device to operate by discharging current from both terminals of the capacitor using only n‐channel metal‐oxide‐semiconductor (NMOS). A table comparing our 6T1C structure with other capacitor‐based charge storage synapses^[^ ^16^, ^23^, ^24^ ^]^ can be found in Section S1 (Supporting Information). We fabricated a single device and 5×5 crossbar array on an 8‐inch silicon wafer and examined weight update, retention, and cycling endurance characteristics. We also experimentally demonstrated parallel on‐chip training operation through linear regression on a crossbar array.

In addition, we developed novel neural network training schemes by co‐optimizing the device and algorithm. The 6T1C synaptic device with standard bias conditions can provide sufficient linearity and symmetry needed for the conventional stochastic gradient descent (SGD) training algorithm. Furthermore, by simply changing bias conditions, the linearity and symmetry of the 6T1C synaptic device can be tailored for the symmetry‐centric Tiki‐Taka algorithm (TTv1),^[^ ^25^ ^]^ we also developed a retention‐centric Tiki‐Taka algorithm (rTT) to efficiently transfer volatile weights of the 6T1C device to average non‐volatile memories so that deep neural networks with large datasets requiring synaptic devices with long retention times can be trained efficiently without losing the accuracy. We designed the 6T1C synaptic device such that the capacitor can be accessed from both top and bottom electrodes so that the reference point can be measured efficiently for the rTT. These device‐algorithm co‐optimizations enabled us to demonstrate an MNIST on‐chip training accuracy of over ≈97% in a wide range of retention requirements even when device and circuit variations were included.

## 2 Results and Discussion

### 2.1 Operation Mechanism of the Synaptic Device

**Figure 1:**
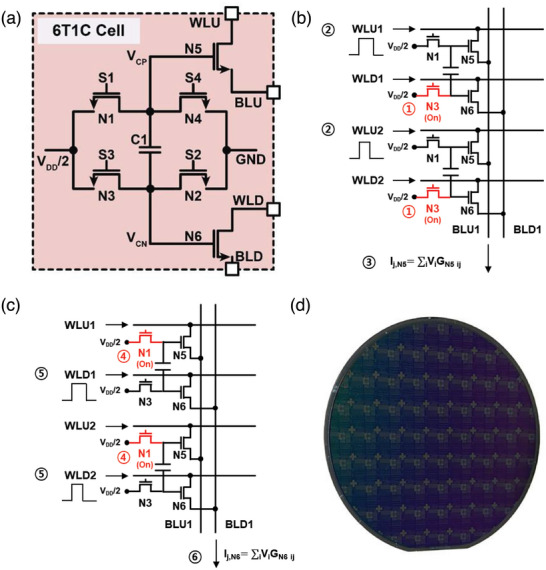
6T1C synaptic device. a) Schematic diagram of the 6T1C synaptic device. A small source‐drain in N5, N6 is essential to operate N5, N6 in the triode region, in which channel resistance depends on the capacitor voltage, *V*_Cap_ (*V*_CP_–*V*_CN_). b) The initial three stages for the feedforward process in 6T1C crossbar array. c)The final three states for the feedforward process in 6T1C crossbar array. The feedforward is concluded by subtracting the ADC values obtained from b and c.d) Photograph of 8‐inch wafers processed with a single synaptic device and crossbar array. The width of the IGZO device channel used for a single synaptic device and crossbar array was 2 µm, and the length was fabricated at the level of 0.5–5 µm.

**Figure** **1**a is a schematic of a synaptic device based on IGZO TFT and capacitor. A single synaptic device is composed of six IGZO TFT and one capacitor (6T1C device). In this design, the capacitor, C_1_, serves as a memory element in the cell and stores the weight value in the form of an electric charge. The two transistors, N5 and N6 serve as a read transistors, and the other four transistors N1–N4 serve to vary the capacitor voltage, *V*_cap_.

For potentiation, a pulse is applied to the N1 to make the upper terminal voltage (*V*_CP_) of the capacitor *V*_dd_/2, and then a pulse is applied to the N2 to increase the voltage of the capacitor *V*_cap_(*V*_CP_–*V*_CN_). For depression, a pulse is applied to the N3 to make the lower terminal voltage (*V*_CN_) of the capacitor *V*_dd_/2, and then a pulse is applied to the N4 to decrease *V*_cap_. In other words, during the on‐chip training process, if pulses are simultaneously applied to N1 and N2, potentiation occurs, while simultaneous pulses applied to N3 and N4 result in depression. It is important to note that in the operation, N1 and N3 as well as N2 and N4, cannot be turned on simultaneously. The voltage change per update pulse is calculated as follows:

(1) $$\Delta V_{\mathrm{cap}}=\frac{i_{N2\left(\mathrm{or}\;N4\right)}\times t_{\mathrm{pw}}}{C}$$

where *i*_N2(or N4)_ is the discharging currents from N2(or N4), respectively, and *t*_pw_ is the pulse width applied to the N2(or N4), and *C* is the capacitance of the 6T1C cell capacitor. To ensure linearity and symmetry characteristics, it is necessary to operate N2 and N4 in the saturation region and discharge a constant current.

The read operation is divided into two separate processes of reading the current flowing through the N5 and N6. For read operation in N5, a pulse is applied to the N3 to make the voltages of the upper and lower terminals of the capacitors *V*_dd_/2 + V_cap_ and *V*_dd_/2, respectively. With *V*_dd_/2 + V_cap_ at the gate terminal of the N5, G_N5_ is measured by applying a small bias between the source and drain of N5. Similarly, a pulse is applied to the N1 to make the voltages of the upper and lower terminals of the capacitors *V*_dd_/2 and *V*_dd_/2–*V*_cap_, respectively. With *V*_dd_/2–*V*_cap_ at the gate terminal of the N6, G_N6_ is measured by applying a small bias between the source and drain of N6. To represent both positive and negative weights, the weight of single 6T1C device, *W*_ij_, is defined by subtracting *G*_N5 _and *G*_N6 _.

(2) $$W_{\mathrm{ij}}=G_{N5\;\mathrm{ij}}-G_{N6\;\mathrm{ij}}$$

Figure 1b,c shows the operation of the 6T1C device crossbar array. For feedforward operation, as shown in Figure 1b, all N3 devices within the crossbar array are initially turned on, setting the capacitor's top and bottom nodes to *V*_DD_/2 + *V*_cap_ and *V*_DD_/2, respectively. Then, the input data encoded in the form of pulse width, as shown in Figure 1b at circle number 2, is applied to the WLU. Afterward, the summed current flowing through each column via N5 is read through BLU, and this current undergoes analog‐to‐digital conversion (ADC). Similarly, to read the current through N6, the N1 devices within the crossbar array are activated. The N1 transistors are turned on, thereby setting the top and bottom nodes of the capacitor to *V*_DD_/2 and *V*_DD_/2–*V*_cap_, respectively. The same pulse width that was applied to WLU is also applied to WLD, and the summed current per column is read through BLD. The read current is then subjected to ADC. Finally, the feedforward process concludes by subtracting the ADC values of the current flowing through N5 and the current flowing through N6.

During the backpropagation process, the roles of WL(WLU, WLD) and BL(BLU, BLD) are reversed compared to their roles during forward inference. The error values encoded in pulse width form are applied to BL(BLU, BLD) direction, and the summed currents along the rows are read. Similarly, to the feedforward process, the currents flowing through N5 and N6 are sequentially read. The values obtained from these two processes are used to stochastically apply pulses to the gates of N1‐N4 transistors. Finally, the weight update is performed based on these pulses.

We fabricated a 6T1C single synaptic device and 5×5 crossbar array on an 8‐inch silicon wafer as shown in Figure 1d. Details of the fabrication can be found in the Experimental Section and essential electrical characteristics of IGZO TFTs can be found in Section S2 (Supporting Information).

### 2.2 Various Properties of a Single Device

**Figure 2:**
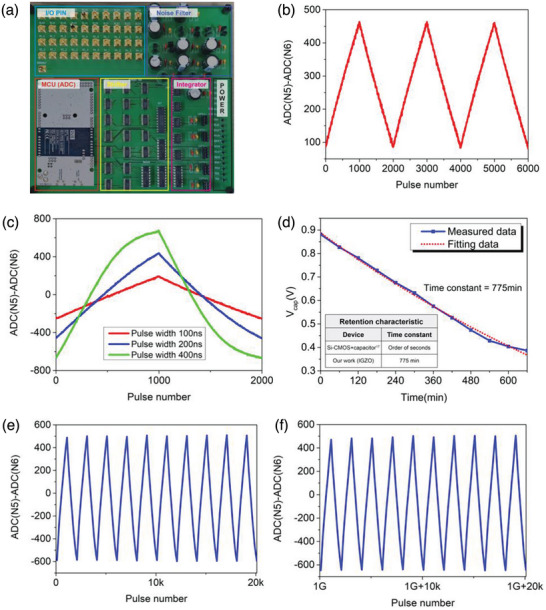
a) PCB photograph with synaptic device measurement b) Weight update result of 6T1C single device (*V*_dd_/2 = 2.0 V). 300 ns 1000 up/down pulses of *V*_ov_ (= *V*_gs_−*V*_th_) = 0.25 V were applied to N2, N4 transistors. c) Weight update curve according to N2, N4 pulse width (*V*_dd_/2 = 1.5 V) 1000 up/down pulse of *V*_ov_ = 1.4 V were applied to N2, N4. d) Results of retention characteristics of 6T1C single device. An off voltage of −2 V was applied to each transistor(N1–N4) e) Measurement results in the initial cycle for measuring cycling endurance of 6T1C f) Measurement result after applying 10^9^ update pulses (update pulse height was 0.5 V/−2 V and length was 1 µs.)

We examined the various characteristics of a single synaptic device. We created a printed circuit board (PCB) combining a microcontroller unit (MCU) and discrete integrated circuit components to interact with an array of synaptic cells. (**Figure** **2**a) Details of PCB can be found in Section S3 (Supporting Information). The synaptic cell current is measured by the current integrator and the ADC on the PCB.

#### 2.2.1 Weight Update

Figure 2b,c shows the weight update characteristics of a 6T1C single device measured with the PCB. Figure 2b shows the measured change of ADC value of a single cell, by applying four cycles of 1000 positive updates followed by 1000 negative updates. To obtain good linearity and

symmetry characteristics of the device by keeping the N2 and N4 in the saturation region, a low overdrive voltage (*V*_gs_–*V*_th_) was intentionally applied to the N2 and N4, and a high voltage was also applied to *V*_dd_/2. We also demonstrate conductance modulation with voltage pulses from 100 to 400 ns (Figure 2c). The resulting curves (Figure 2b,c) show that the 6T1C device exhibits very linear and symmetric weight update characteristics with 1000 conductance states and conductance modulation of the device can be accurately controlled by changing the measurement condition in the same device.

#### 2.2.2 Retention Characteristics

Figure 2d shows the retention measurement of a 6T1C single device. To evaluate the retention characteristics of the device, first, the capacitor was intentionally charged by repeatedly applying potentiation pulses to the synaptic cell. Then, we apply an off voltage to all transistors(N1–N4) and perform read operations at every predetermined time interval (60 min). After converting the measured ADC value into capacitor voltage, exponential fitting was performed to extract the time constant, and as a result, 775 min were obtained. These results indicate that the 6T1C device exhibits very good retention characteristics compared to the conventional silicon and capacitor‐based synaptic devices with a time constant on the order of seconds.^[^ ^17^ ^]^

#### 2.2.3 Cycling Endurance

Another important factor that affects deep neural network training based on crossbar array technology is the endurance characteristics of synaptic devices. Resistive switching devices, such as RRAM, have been reported to demonstrate an endurance of ≈10^5^–10^7^ cycles, wherein they distinguish only between the high‐resistance state (HRS) and low‐resistance state (LRS) at the array level.^[^ ^26^, ^27^, ^28^, ^29^ ^]^ In contrast, the charge storage synaptic device based on Si‐CMOS and capacitor exhibits a semi‐infinite endurance characteristic.^[^ ^17^ ^]^ In our study, we validated the endurance characteristics of IGZO TFT‐based 6T1C devices.

Figure 2e,f shows the endurance characteristics of a 6T1C single device. An update pulse of 10^9^ was applied by repeating one cycle of 1000 up/1000 down pulses 500 000 times to the synaptic device. By comparing the output ADC value of the initial cycle and the last cycle shown in Figure 2e,f, respectively, it was shown that the device is still working even after 10^9^ pulses are applied and the output range of ADC value hardly changes, which confirms that the 6T1C synaptic device not only still survives but also a stable analog characteristic over a large number of cycles. In addition, through the optimization of bias conditions that minimize negative bias stress (NBS) and positive bias stress (PBS),^[^ ^30^ ^]^ which induce changes in the characteristics of the transistors within the 6T1C device, it is possible to expect the achievement of endurance characteristics approaching semi‐infinity.

### 2.3 Implementation of Linear Regression on a Crossbar Array

**Figure 3:**
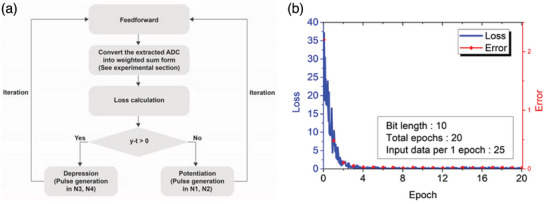
a) Flow chart for linear regression training. Training consists of two steps: feedforward and weight update. Loss is defined as $\frac{1}{2}{\left(y-t\right)}^{2}$ using MSE function. b) Evolution of loss and error throughout the training. Both loss and error converge to 0 as training progresses, and the inset of b) lists parameters and relevant numbers used in this demonstration. 25 input data sets are trained at each epoch. During the demonstration, the bit length was set to 10 and the learning rate was 0.05. The error in Figure 3b is defined as $\sum_{n\;=\;1}^{5}{\left(w_{n}-t_{n}\right)}^{2}$.

With a 6T1C 5×1 crossbar array, we experimentally conducted linear regression to evaluate the on‐chip training performance of the synaptic device. The learning process is summarized in **Figure** **3**a, and input data in the feedforward process and stochastic update pulse in the weight update process were generated in real‐time through MCU located on PCB. First, we generated an input dataset for the feedforward process, and this was applied to the word lines (WLs) of five synaptic devices in the form of pulse width, respectively.

The input data matrix is composed of four randomly generated data and one fixed value serving as a *y*‐intercept in the form of *x*_i_ =  [*x*_1_,*x*_2_,*x*_3_,*x*_4_,*a*]. Once the feedforward process is carried out, a value of *y* in Equation (3) is generated.

(3) $$y=x_{i}\cdot {\left[w_{1},w_{2},w_{3},w_{4},w_{5}\right]}^{T}$$

where *x_i_* denotes the input data matrix and [*w*_1_,*w*_2_,*w*_3_,*w*_4_,*w*_5_] denotes the weight matrix. Then *y* is compared with the target value *t* in Equation (4) to generate the error value δ in Equation (5).

(4) $$t=x_{i}\cdot {\left[t_{1},t_{2},t_{3},t_{4},t_{5}\right]}^{T}$$

(5) $$\delta =x_{i}\cdot {\left[w_{1},w_{2},w_{3},w_{4},w_{5}\right]}^{T}-t$$

where [*t*_1_,*t*_2_,*t*_3_,*t*_4_,*t*_5_] denotes the target weight matrix. The process of converting the output ADC value into weighted sum and loss calculation was performed through the MCU located on PCB (See the Experimental section for linear regression details). Then, via the stochastic update scheme,^[^ ^31^ ^]^ a weight update is performed. The weight update amount of each synaptic cell follows Equations (6) and (7).

(6) $$\Delta w_{n}=-\eta x_{n}\delta \left(n=1-4\right)$$

(7) $$\Delta w_{5}=-\eta a\delta$$

where *x*_n_ and *a* denote the input data applied to each synaptic cell, δ denote errors extracted through feedforward, η denotes the learning rate. In the weight update process, *x*_n_ and *a* were translated as N1 or N3 pulse generation probability and δ was translated into N2 or N4 pulse generation probability. As shown in Figure 3a, the sign of (*y* − *t*) will determine whether to generate pulses for potentiation or depression. By repeating this procedure, we can train the 6T1C 5×1 crossbar array and solve the linear regression problem. Figure 3b shows that the loss and error converge to zero as training proceeds. These linear regression results demonstrate the automatic and parallel on‐chip training capability of the 6T1C array.

### 2.4 Algorithm Optimization for 6T1C Device: Retention‐Centric Tiki‐Taka

The achievement of the AiMC system came from both algorithm‐level and hardware‐level approaches, aiming for enhanced optimization through hardware‐algorithm co‐design. Therefore, we conducted simulations by applying our device to various learning algorithms: conventional SGD algorithm and TTv1.^[^ ^25^ ^]^ The TTv1 is a recently proposed algorithm to overcome the stringent symmetry requirement of analog synaptic devices and several improved versions such as TTv2 and c‐TTv2^[^ ^32^, ^33^ ^]^ have been additionally proposed to overcome limitations such as read noise and number of states. In this study, we conducted the simulations focusing on TTv1, the most fundamental of them. Our simulation results demonstrated that our device is well suited for TTv1 as well as the SGD algorithm. In addition, we devised a new robust algorithm specialized for devices, rTT, that goes beyond TTv1. This more robust algorithm 1) shows no decrease in learning accuracy even when the retention level required for learning increases and 2) can set the reference conductance easily on the device itself without a separate reference cell array utilizing the structural characteristics of 6T1C.

First, we simulated neural network training with the SGD algorithm based on the weight update characteristics and retention time from measurement results shown in Figure 2b,d. In addition, device variations of 6T1C, a variation of 15% were applied to NL, which is the parameter representing device asymmetry, 7% to *G*_max_ and *G*_min_, 6% to Δ*w*_min_, 15% to retention time, and 15% to G_leak_. *G*_max_ and *G*_min_ denote the maximum and minimum conductance of the device, respectively. G_leak_ denotes conductance in which the volatile device converges after complete retention failure. In the case of cycle standard deviation, 5% standard deviation was applied to write noise, 6% to current sum, and 30% to Δ*w*_min_. The following variations were applied to all simulations other than the SGD algorithm. Assuming the training cycle length per layer (forward + backward + update) is 200 ns,^[^ ^17^ ^]^ ≈98.5% accuracy was obtained. (See Section S5, Supporting Information) This result is attributed to the symmetrical behavior and good retention characteristics of the 6T1C synaptic device.

**Figure 4:**
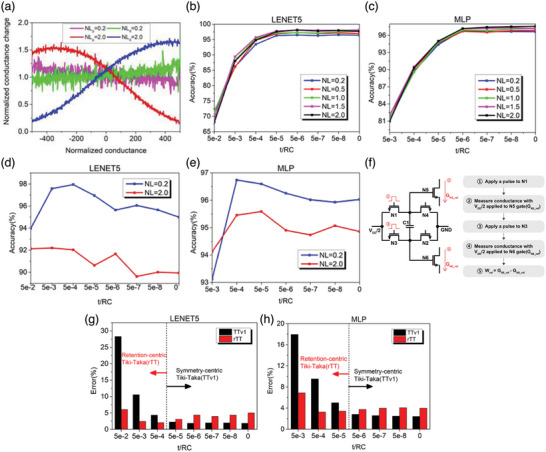
a) Conductance–conductance change form of results obtained under various measurement conditions. In the figure, NLP and NLD represent non‐linearity observed during the process of potentiation and depression, respectively. The equations related to these concepts can be found in Equations (8) and (9). NL = 0.2 and 2.0 are the results of converting Figure 2b and the green line of Figure 2c, respectively. In the case of NL = 2.0, linear regression analysis was used only for some domains for learning. (Figure S6, Supporting Information) Result of applying TTv1 by reflecting various values within the NL range of 6T1C device in (b) LENET5 and c) MLP (see Tables S4 and S5, Supporting Information, for the accurate learning accuracy value) Neural network training results by applying the rTT to NL = 0.2, 2.0 in (e) LENET5 and f) MLP (see Tables S6 and S7, Supporting Information, for the learning accuracy results according to the detailed NL values) Because a fixed learning rate was used for all retention ranges, the accuracy decreased in 5e‐2 of LENET5 and 5e‐3 of MLP. However, when the optimized learning rate was used for training in 5e‐2 of LENET5 and 5e‐3 of MLP, an accuracy of ≈97% was achieved. (Figure S8, Supporting Information) f) Method of setting reference conductance in rTT. Since the reference conductance in rTT means the conductance when *V*_Cap_ = 0, it is read as the difference between the current of N5 with a pulse applied to N1 and the current of N6 with a pulse applied to N3. g,h) Learning results of applying TTv1 and rTT at various retention levels in LENET5, and MLP respectively. In Figure 4 g,f, only the best case among several NL values was shown for TTv1, and the case where NL = 0.2 for rTT was shown.

Second, we conducted neural network training with the TTv1, recently developed training algorithms designed for asymmetric analog synaptic devices. The TTv1 operates fully in parallel and trains the core device through the update information obtained from the auxiliary device. In this case, we use the 6T1C device, which has excellent update characteristics but fundamentally has leakage, as the auxiliary device, and average non‐volatile memory (NVM) as the core device to periodically transfer the weight of 6T1C to NVM so that it could be read without loss of weight during the inference process. To utilize the TTv1 for training, the weight update measurement results of 6T1C were converted to the conductance–conductance change form of **Figure** **4**a. Then Figure 4a was modeled via linear regression analysis to extract simulation parameters in Equations (8) and (9). (See Section S6, Supporting Information)

(8) $$\Delta G_{p}=\left(1-NL_{p}\times \frac{G-G_{\mathrm{sym}}}{G_{\max}-G_{\min}}\right)\times \Delta G_{\mathrm{sym}}$$

(9) $$\Delta \;G_{d}=\left(1-NL_{d}\times \frac{G-G_{\mathrm{sym}}}{G_{\max}-G_{\min}}\right)\times \Delta G_{\mathrm{sym}}$$

where Δ*G*_p_, Δ*G*_d_ denote the conductance change in one potentiation, depression pulse, respectively; *G*_sym_ denotes the symmetric conductance that satisfies Δ *G*_p_ =  Δ*G*_d_. As shown in Figure 4a, it was possible to extract both highly linear update results (NL ≈ 0.2) and intentionally asymmetric update results (NL ≈ 2.0) by changing the measurement conditions in the same device. Previous studies have shown that the NL combination of core and auxiliary devices is important to achieve optimal learning accuracy using TTv1.^[^ ^34^ ^]^ In other words, it is important to obtain a target NL value to find the optimal combination. However, in a typical resistive switching device, it is impossible to obtain a target NL value because the conductance modulation mechanism relies on a random process at the atomic level.^[^ ^14^ ^]^ On the other hand, as shown in Figure 4a, our device can have a wide range between NL = 0.2–2.0 by changing the measurement condition in the same device, and the target NL value can be easily obtained based on a clear conductance modulation mechanism. Figure 4b,c shows the result of applying the TTv1 under the same core device condition by reflecting various values within the NL range of the 6T1C device in the LENET5 and multi‐layer perceptron (MLP) neural network structures, respectively. As in the results of previous studies,^[^ ^34^ ^]^ the learning accuracy was changed according to the NL of the 6T1C device. However, our device can easily obtain the target NL by changing the measurement conditions, so it is a device that can achieve optimal learning accuracy through the TTv1 regardless of the type of core device.

In addition, by using the TTv1 for the 6T1C device, weight transfer can also be performed. Despite the good retention characteristics of 6T1C devices, the weight transfer process that conveys the stored weights to NVM is essential because of long‐term retention loss. However, the weight transfer technique requires serial access to cross‐point elements either one‐by‐one or row‐by‐row.^[^ ^8^, ^35^, ^36^ ^]^ Naive weight transfer can cause tremendous overhead for large networks because it involves time‐consuming serial operations and repeatedly programming and verifying weights.^[^ ^37^, ^38^ ^]^ On the other hand, by using the TTv1, learning and weight transfer can be performed simultaneously and fully in parallel.

Although the 6T1C device proved to be a well‐suited device for the TTv1, the learning accuracy deteriorated as the time required for learning increased as shown in Figure 4b,c. Therefore, we proposed a new algorithm, the rTT to recover the learning accuracy even when longer retention is required. First, we analyzed the phenomenon that the accuracy decreases according to retention in the TTv1 by adapting the formula proposed in the previous study.^[^ ^39^ ^]^

When the TTv1 is performed using a volatile device as an auxiliary device and a non‐volatile device as a core device, the weight update aspect of the auxiliary device and the core device follows Equations (10) and (11), respectively.

(10) $$\begin{array}{ccc} \dot{A} & = & -\left(1-e^{-\frac{t_{\mathrm{update}}}{RC}}\right)\left(A-A_{\mathrm{leak}}\right)-\eta_{A}e^{-\frac{t_{\mathrm{update}}}{RC}}\left(\left(\frac{\partial E}{\partial C}+\varepsilon \left(t\right)\right)\right.\\ & & \left.+\;NL_{A}\frac{A-A_{\mathrm{sym}}}{A_{\max}-A_{\min}}\left|\frac{\partial E}{\partial C}+\varepsilon \left(t\right)\right|\right) \end{array}$$

(11) $$\dot{C}=\eta_{C}\left(A-A_{\mathrm{ref}}\right)-\eta_{C}\left|A-A_{\mathrm{ref}}\right|NL_{C}\frac{C-C_{\mathrm{sym}}}{C_{\max}-C_{\min}}$$

where *t*_update_ denotes the training cycle length per layer in an auxiliary array and ε(*t*) denotes the stochastic effect that occurs during the update. η_A _and η_C_ are the learning rate of the auxiliary and the core device, respectively. *E* denotes cost function.

Then for the weight of the auxiliary and the core device to reach a steady state, Equations (12), and (13) must be satisfied, respectively. In the TTv1 using NVM, the global minimum can be reached because |*A* −  *A*_sym_ =  0| can be satisfied after sufficient learning is carried out by setting *A*_ref_ as *A*_sym_. However, when a volatile device is used as an auxiliary device, the right side of Equation (12) does not converge to 0 and thus the global minimum cannot be reached. As a result, as the required retention time for training increased, the learning accuracy decreased as shown in Figure 4b,c.

(12) $$\begin{array}{ccc} \langle \frac{\partial E}{\partial C}\rangle & = & -\frac{NL_{A}}{A_{\max}-A_{\min}}\left|\frac{\partial E}{\partial C}+\varepsilon \left(t\right)\right|\left(A-A_{\mathrm{sym}}\right)\\ & & -\;\eta_{A}^{-1}\left(e^{\frac{t_{\mathrm{update}}}{RC}}-1\right)\left(A-A_{\mathrm{leak}}\right) \end{array}$$

(13) $$\langle A\rangle -A_{\mathrm{ref}}=NL_{C}\;\frac{C-C_{\mathrm{sym}}}{C_{\max}-C_{\min}}\left|\left(A-A_{\mathrm{ref}}\right)\right|$$

However, the decrease in accuracy due to retention can be solved with a new device‐specific algorithm(rTT) that takes *A*_ref_ as *A*_leak_. Due to the structure of the 6T1C device, the expected values of *A*_sym_ and *A*_leak_ are the same when *V*_cap_ = 0, and the 6T1C device is capable of highly linear weight updates shown in Figure 4a. Therefore, the influence of the term related to asymmetry, which is the first term on the right side of Equation (12), can be almost negligible, and it can be expected the global minimum will be reached if *A*_ref_ is set to *A*_leak_.

Note that as the global minimum is reached, $|\frac{\partial E}{\partial C}+\varepsilon \left(t\right)|$ of Equation (12) becomes smaller thus the effect of asymmetry is further reduced. Figure 4d,e shows the neural network training results by applying the rTT to NL = 0.2, 2.0 in LENET5 and MLP. In the highly linear case with NL = 0.2, an accuracy of ≈97.5% can be achieved even if the required retention time increases. In addition, as analyzed in Equation (10), it was confirmed that the accuracy decreased as the asymmetry of the device increased.

The rTT also has the advantage of being able to set the reference conductance quickly and easily by 6T1C itself without a separate reference cell array. It is known that is important to set an accurate and stable reference conductance as symmetric conductance in the TTv1,^[^ ^39^ ^]^ but the previously proposed symmetric conductance setting method is relatively complex and requires an additional array.^[^ ^40^ ^]^ On the other hand, with the rTT, the 6T1C can take the reference conductance quickly and accurately by performing one more read operation (Figure 4f). In addition, as the characteristics of the device change during training, the symmetric conductance of the device itself or the conductance of the reference device may change, resulting in reduced accuracy. However, for the rTT, the reference conductance can be read stably even if the characteristics of the device change during training.

Figure 4g,h shows the learning results of applying TTv1 and rTT at various retention levels. As confirmed in Equation (12), the retention and asymmetry of the devices had a complex effect on the learning accuracy, and algorithms required for optimal learning differed according to retention levels. However, by using the 6T1C, it is possible to flexibly select the algorithms according to the retention level. If the retention required for learning increases due to complex datasets or neural networks, the optimal accuracy can be obtained by applying the rTT using a highly linear update condition. If the retention of the device is sufficient for learning, the optimal accuracy was obtained by applying TTv1 using an update condition suitable for the asymmetry of the core device.

Furthermore, the 6T1C device and optimized algorithm can also improve scalability, which is a disadvantage of capacitor‐based synapses. As the size of the capacitor decreases, it is difficult to achieve sufficient retention time for learning, so there is a limit to the scalability of the capacitor‐based synaptic device. For example, it is known to require a large capacitance of 100fF capacitance/cell for a CNN.^[^ ^17^ ^]^ However, since the IGZO‐based 6T1C device has a much smaller leakage current and can apply an algorithm robust to retention, capacitor size can be reduced, thus device scalability can be improved. For example, applying an IGZO TFT^[^ ^22^ ^]^ and capacitor^[^ ^41^ ^]^ with the lowest current level reported so far, high learning accuracy can be achieved by training a large input data with a synapse with a 10fF capacitance/cell. (See Section S8, Supporting Information) Therefore, the 6T1C device is a versatile and practical device that can be applied to large input data and complex neural networks.

## 3 Conclusion

We have reported a novel synaptic device using IGZO TFT with low leakage current and capacitor to solve the retention problem in capacitor‐based charge storage synapses. By fabricating a single synaptic device and a 5×5 crossbar array, we demonstrate that our novel device can provide not only linear and symmetric weight update but also sufficient retention time and parallel on‐chip training operations. We also demonstrated the importance of co‐optimization of the device‐algorithm by developing an efficient yet realistic training algorithm to compensate for remaining device non‐idealities such as drifting reference and long‐term retention loss. Our novel algorithm does not require a separate reference cell array and could reach a high learning accuracy of ≈97% even when the retention time required for training increases, enabling smaller synaptic array sizes with smaller capacitors. We expect that the size of the 6T1C device can be further reduced with ultralow‐leakage and nanoscale IGZO TFTs and capacitors that have previously been reported. The device footprint can be further improved through Monolithic 3D (M3D) integration^[^ ^41^, ^42^, ^43^ ^]^ and vertical channel thin‐film transistors (VTFTs) based on IGZO atomic layer deposition (ALD).^[^ ^44^, ^45^ ^]^ Therefore, we believe that our 6T1C device is a practical synaptic device for neuromorphic computing.

## 4 Experimental Section

### Device Fabrication

The synaptic array structure composed of the IGZO TFT and capacitor used in this study was made of a 4‐metal, 8‐metal layer on top of silicon oxide formed on a silicon base. First, a 200 Å thick tungsten metal to be used as the capacitor's lower electrode was deposited. A rectangular lower electrode with various widths and its connecting wiring was patterned using photolithography and dry etching. Afterward, a high‐k oxide to be used as a capacitor insulator was deposited to the required thickness by an ALD. Note that a capacitor using an appropriate high‐k material must be fabricated to satisfy the capacity and leakage current requirements of the synaptic capacitor. VIA for connecting the lower electrode and the upper electrode was formed by a wet etching after photolithography only at the point where it intersects the wiring connected to the upper electrode in a region separate from the capacitor. Next, 200 Å thick tungsten metal to be used as the upper electrode of the capacitor was deposited by CVD. A rectangular upper electrode and connecting wiring having various widths were patterned in consideration of the shape in which the lower electrode was formed. Next, an appropriate oxide used as an IGZO TFT underlayer was deposited. The VIA process for connecting the upper electrode and the metal used as the source/drain of the TFT was performed by photolithography and wet etching. After that, 200 Å tungsten metal to be used as the source/drain of the IGZO TFT was deposited, patterned, and etched. IGZO, the channel material of IGZO TFT, was deposited to a thickness of 100 Å in 2.44 W cm^−2^ RF bias plasma in 1 Pa Ar/O_2_ atmosphere using a sputtering facility equipped with a target composed of In:Ga:Zn = 1:1:1. Then, it was formed on the channel site through photolithography and wet etching. A high‐k material to be used as the gate oxide of the IGZO TFT was deposited. Note that the upper and lower high‐k material adjacent to the IGZO channel must be considered to satisfy the requirements for the on/off ratio and leakage current characteristics of the IGZO TFT. VIA patterning and etching were performed at the site where the connection between the source/drain and the upper electrode was required. After that, a tungsten metal to be used as the upper gate electrode of the IGZO TFT was deposited, and the synaptic structure was completed by dry etching after photolithography.

### Experimental Setup for Device Measurement

The peripheral circuit for synapse measurement was implemented on the PCB combining MCU and discrete devices. The synaptic cell on an 8‐inch wafer was contacted with a 45‐pin probe card mounted on an Eg4090, and the voltage to be applied to each synaptic cell was applied to the PCB through the DC power supply(2230g‐30‐3). Personal computer(PC) and MCU communicate with universal asynchronous receiver‐transmitter (UART), and input signals such as pulse width and repetition number were input from PC. Supplementary Figure 2 shows the connection structure of the PC, MCU, and PCB. The PCB mode for synaptic device measurement was divided into feedforward, backpropagation, and weight update, and the data flow for each mode and discrete device information used in PCB are all shown in Section S 3(Supporting Information). In the case of read and update pulses, the measurement was performed by giving a pulse in microseconds unit considering the settling time of the MCU, but when a pulse of nanoseconds was applied, the pulse of the MCU was used as a trigger and a pulse in nanoseconds unit was applied using a pulse generator (81110A, 81150A).

### Linear Regression

In linear regression, when feedforward was conducted, the output was in the form of ADC. Therefore, it was essential to convert the output ADC into a weighted sum for loss calculation. The weighted sum was defined as follows:

(14) $$\sum_{n\;=\;1}^{5}x_{n}\times w_{n}=\sum_{n=1}^{5}\frac{x_{n}}{x_{\max}}\times 2\left(\frac{G_{n}-G_{\mathrm{ref}}}{G_{\max}-G_{\min}}\right)\times x_{\max}$$

where *G*_max_ and *G*_min_ denote the maximum and minimum conductance of the device, respectively, and *G*_ref_ denotes the conductance when weight is 0; *x*_max_ denotes the maximum value among input data within a predetermined range, and *x*_n_ denotes the current input data applied to each cell. The $\frac{x}{x_{\max}}\times x_{\max}$ part in Equation (14) means input data, $2(\frac{G_{n}-G_{\mathrm{ref}}}{G_{\max}-G_{\min}})$ means the normalized weight in the range of −1 to 1 of each cell, and the product of the two components means the input × weight, which is a weighted sum. Then, the right side of Equation (14) can be rewritten as

(15) $$\begin{array}{ccc} \sum_{n\;=\;1}^{5}\frac{x_{n}}{x_{\max}}\times 2\left(\frac{G_{n}-G_{\mathrm{ref}}}{G_{\max}-G_{\min}}\right)\times x_{\max} & = & 2\left(\frac{ADC_{\mathrm{feedforward}}-ADC_{\mathrm{ref}}}{ADC_{\max}-ADC_{\min}}\right)\\ & & \times \;x_{\max} \end{array}$$

where *ADC*_feedforwad_ denotes the ADC extracted through the feedforward process and *ADC*_max_ is the ADC extracted when the maximum value(*x*_max_) of the input data is applied when the conductance of the synaptic cell is maximum(when the voltage stored in the capacitor is *V*_dd_/2); *ADC*_min_ is the ADC extracted when the maximum value (*x*_max_) of the input data is applied when the conductance of the synaptic cell is minimum(when the voltage stored in the capacitor is −*V*_dd_/2). To determine *ADC*_max_, *ADC*_min_, *ADC*_ref_ in Equation (15), ADC was extracted for five cells before training, and *ADC*_max_, *ADC*_min_, *ADC*_ref_ were determined as the average of the five values obtained.

### MNIST Pattern Recognition Simulation using TTv1 with 6T1C Device

To apply the Tiki‐Taka algorithm using the 6T1C device, IBM Analog Hardware Acceleration^[^ ^46^ ^]^ Kit ver.0.5.1 was used. First, the linearStepDevice was modified to reflect the characteristics of the 6T1C device. Second, the TransferDevice was modified to apply the rTT algorithm. During ANN simulation, *G*_leak_ of 6T1C device follows *N*(*G*_sym_,(0.15*G_sym_)^2^), and a cycle‐to‐cycle standard deviation of 30% was used for every update. NL values of 6T1C, 0.2, 0.5, 1.0, 1.5, and 2.0 were swept, and in each case, the device‐to‐device standard deviation of 15% of the NL value was applied. Since the number of states of 6T1C can be >10 bits, Δ *w*_min_ =  0.002(≈1000 steps), and Δ*w*_min_ device‐to‐device 6% was set. The weight leakage phenomenon of the auxiliary device was reflected just before updating the core device, and 15% device‐to‐device std was applied for retention. In the case of rTT, the initialization of the auxiliary device was set to *G*_leak_, which was an initialization that was set naturally without applying any pulse to the device. As the core device, a virtual NVM with characteristics of Δ *w*_min_ =  0.02(≈100 steps), Δ*w*_min_ device‐to‐device std 10%, NL = 1.8, NL device‐to‐device std 15%, and update cycle‐to‐cycle std 30% was used. When applying the TTv1, feedforward and backpropagation were performed only with the core device by setting gamma to 0 in W  =  γA + C,^[^ ^25^ ^]^ and the update was performed on the auxiliary device(6T1C). The MLP neural network structure of the simulation consisted of 784 input neurons, 256, 128 hidden neurons, and ten output neurons, and a sigmoid function was used for the activation function between each layer. The logsoftmax classifier was applied to the output layer. up to 50 epochs were trained with a mini batch of one image, and the average accuracy of the last five epochs was used in Figure 4. For the LENET5 structure of the simulation, 28 ×  28 ×  1 image input, 16 conv1 5 ×  5 ×  1 kernel – hyperbolic tangent – maxpool – 32 conv2 5 ×  5 ×  16 kernel – hyperbolic tangent – maxpool – hyperbolic tangent – FC 512 × 256 × 10 – logsoftmax neural network structure was used. Up to 30 epochs were trained with a mini batch of four images, and the accuracy of the last five epochs was used in Figure 4. In addition, the weight updates of the devices were made to occur when the stochastically generated pulses based on the input and backpropagated values overlap at the same time according to the stochastic update scheme.^[^ ^26^ ^]^

## Conflict of Interest

The authors declare no conflict of interest.

## Supporting information

Supporting Information

## Data Availability

The data that support the findings of this study are available from the corresponding author upon reasonable request.
