# Supplementary material for: Device‐Algorithm Co‐Optimization for an On‐Chip Trainable Capacitor‐Based Synaptic Device with IGZO TFT and Retention‐Centric Tiki‐Taka Algorithm
Source: Adv Sci (Weinh). 2023 Aug 9;10(29):2303018. doi: 10.1002/advs.202303018 (PMC10582414; doi:10.1002/advs.202303018)
Supplement: Supplementary file 1 — Supporting Information [file ADVS-10-2303018-s001.pdf]

ADVANCED SCIENCE
Open Access

Supporting Information

for *Adv. Sci.*, DOI 10.1002/advs.202303018

Device-Algorithm Co-Optimization for an On-Chip Trainable Capacitor-Based Synaptic Device with IGZO TFT and Retention-Centric Tiki-Taka Algorithm

Jongun Won, Jaehyeon Kang, Sangjun Hong, Narae Han, Minseung Kang, Yeaji Park, Youngchae Roh, Hyeong Jun Seo, Changhoon Joe, Ung Cho, Minil Kang, Minseong Um, Kwang-Hee Lee, Jee-Eun Yang, Moonil Jung, Hyung-Min Lee, Saeroonter Oh*, Sangwook Kim* and Sangbum Kim**

Supporting Information

Title Device-algorithm co-optimization for an on-chip trainable capacitor-based synaptic device with IGZO TFT and retention-centric Tiki-Taka algorithm

Jongun Won, Jaehyeon Kang, Sanujun Hong, Narae Han, Minseung Kang, Yeaji Park, Youngchae Rok, Hyeongjun Seo, Changhoon Joe, Ung Cho, Minil Kang, Minseong Um, Kwang-Hee Lee, Jee-Eun Yang, Moonil Jung, Hyung-Min Lee, Saeroonter Oh*, Sangwook Kim*, and Sangbum Kim**

S1. Comparison of 6T1C structure with other charge storage synaptic devices

	3T1C[1]	2T1C[2]	2T0C[3]	6T1C
Device structure	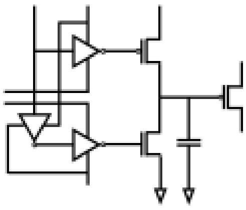	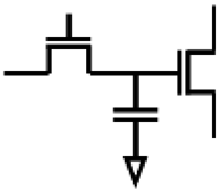	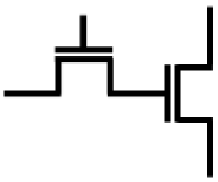	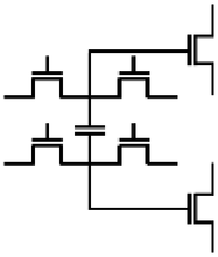
Device component	PMOS, NMOS, Capacitor	IGZO TFT, Capacitor	IGZO TFT	IGZO TFT, Capacitor
Pros	Linearity & Symmetry, On-chip trainable	Device footprint, Retention charateristics	Retention charateristics	Linearity & Symmetry, Retention charateristics, On-chip trainable, Optimized for Tiki-Taka
Cons	Retention charateristics, Logic circuit required for on-chip training	On-chip training is not feasible	Additional circuits required for on-chip training, Device-to-device variation	Device footprint

Table S1. Pros and Cons of 6T1C device and other charge storage synaptic device

Before the 6T1C device research, several synaptic device researches based on transistors and capacitors have been studied. In 2017, a highly promising on-chip trainable 3T1C synaptic device, utilizing Si-CMOS technology and capacitors, was developed, demonstrating exceptional linearity and symmetry characteristics.[1] However, the 3T1C devices not only require additional logic circuits for on-chip training but also have a retention problem due to the large leakage current of Si-CMOS. To solve the retention problem that occurs in Si-CMOS-based devices, research on synapses combining IGZO TFTs with low leakage current and capacitor has been conducted. However, since the viable p-type metal oxide TFT does not exist, linearity and symmetry of weight update, a major requirement of on-chip trainable synaptic devices, has been difficult to achieve. Due to the aforementioned limitations, the 2T1C device that was designed in 2020, utilizing IGZO TFTs and capacitors, was limited to performing only inference tasks, as it was unable to support on-chip training capabilities.[2] In a study reported in 2022, it was found that employing two n-type IGZO TFTs (2T0C) enabled the achievement of desirable linearity and symmetry.[3] However, it is important to note that the linearity and symmetry characteristics in this work were accidentally achieved due to the unexpected alignment of the non-ideal elements within the two devices. Therefore, there is no guarantee that a large array can be operated under identical conditions, and as a result, the device variation characteristics are not favorable. Furthermore, the 2T0C structure is unable to support on-chip training without the aid of additional complex peripheral circuits. On the other hand, the 6T1C device can achieve linearity and symmetry characteristics by utilizing only n-type TFTs, and it exhibits exceptional retention properties attributed to the remarkably low leakage current characteristics of IGZO TFTs. Moreover, as demonstrated in the main manuscript, the 6T1C device allows for easy modulation of weight update tendencies, making it highly suitable not only for the SGD algorithm but also for the Tiki-Taka algorithm. The remaining issues associated with the 6T1C device were shown to be solvable through the utilization of algorithms specifically optimized for the device. Although the use of 6 IGZO TFTs in the 6T1C device presents a drawback in terms of device footprint compared to other devices, the 6T1C architecture enables on-chip training capabilities without the need for additional circuits. In addition, it is possible to further improve the device footprint through processes such as Monolithic 3D(M3D) integration.[4] Therefore, we believe that the 6T1C device, which utilizes only n-type IGZO TFTs, is an extremely practical device for neuromorphic computing.

S2. Essential electrical characteristics of IGZO TFT

Essential electrical characteristics of IGZO TFT	
**Parameters**	**Values**
Channel length(μm)	5
Channel width(μm)	2
TFT Vth(V)	Median: 0.14 St dev: 0.06
TFT on-current(A)	Median: 3.3×10^{-7} St dev: 8.7×10^{-8}
Mobility(cm^2/V·s)	Median: 3.70 St dev:0.48
Sub-threshold swing(mV/dec)	Median: 82.5 St dev: 4.1

Table S2. Essential electrical characteristics of IGZO TFTs measured on 21 dies on an 8-inch wafer. The TFT on-current in the table is the value measured at V_{ds}=1.5V and V_{gs}=1V. The TFT V_{th} in the table corresponds to the voltage at which $I_d=10^{-10}$A.

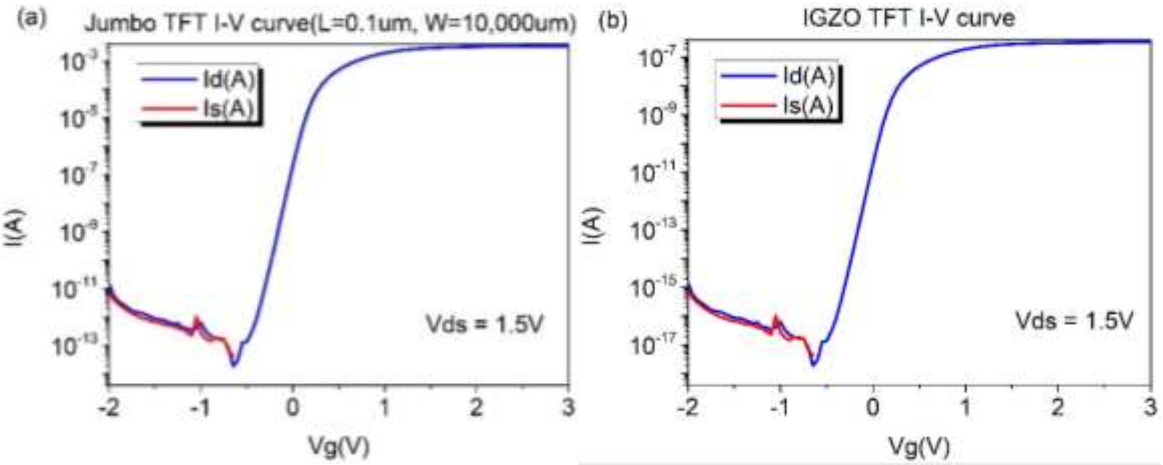

Figure S1. Leakage current characteristics of IGZO TFT.

To verify the leakage current characteristics of IGZO TFT, a specialized measurement method is necessary for the IGZO TFT as its leakage current is below the detection limit(0.1pA) of the conventional current measurement method. Therefore, we intentionally fabricated a large-sized TFT(Jumbo TFT) with a channel width of 10,000 μm to precisely measure the leakage current, which tends to increase proportionally with the channel width. Figure S1-(a) shows the I-V curve of Jumbo TFT. Subsequently, we performed calculations to determine the leakage current per channel width of 1μm. As shown in Figure S1-(b), the obtained leakage current was approximately 10^{-17}A/μm. Considering the reported levels of minimum leakage current in IGZO TFTs[5], a substantial reduction in leakage current is anticipated, leading to enhanced retention charateristics. Consequently, the 6T1C device holds great promise as a favorable and highly promising device for neuromorphic computing.

S3. Behavioral Mechanism of synaptic measurement PCB

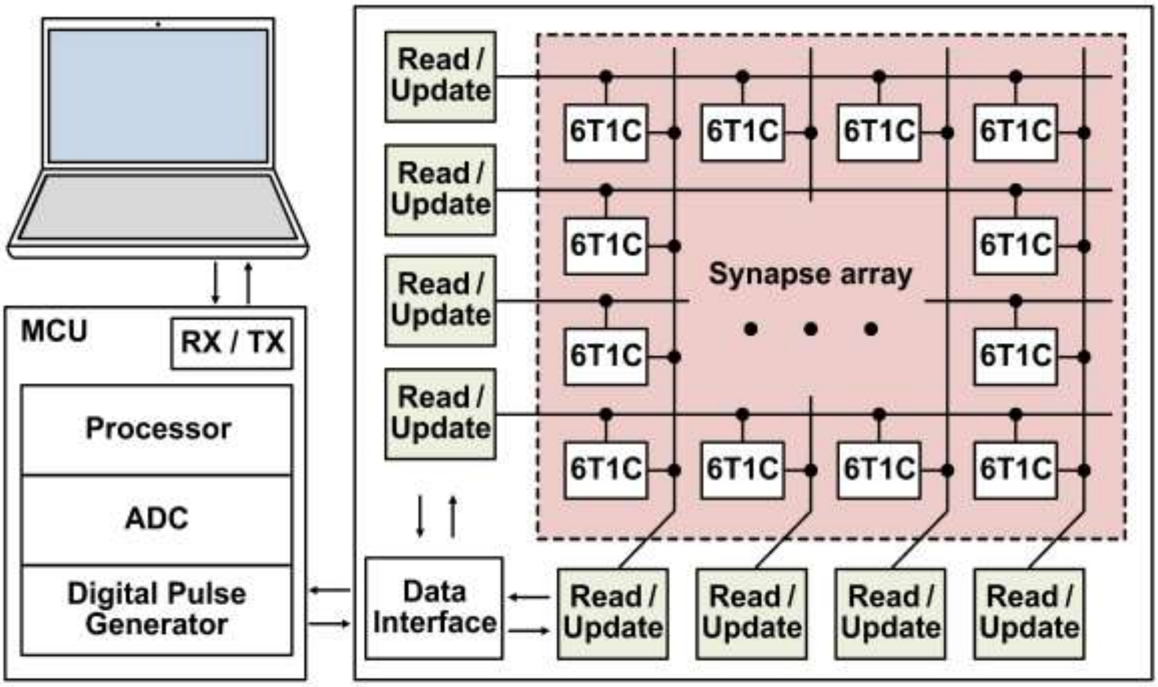

Figure S2. Block diagram of the neuromorphic system with 6T1C synapse array

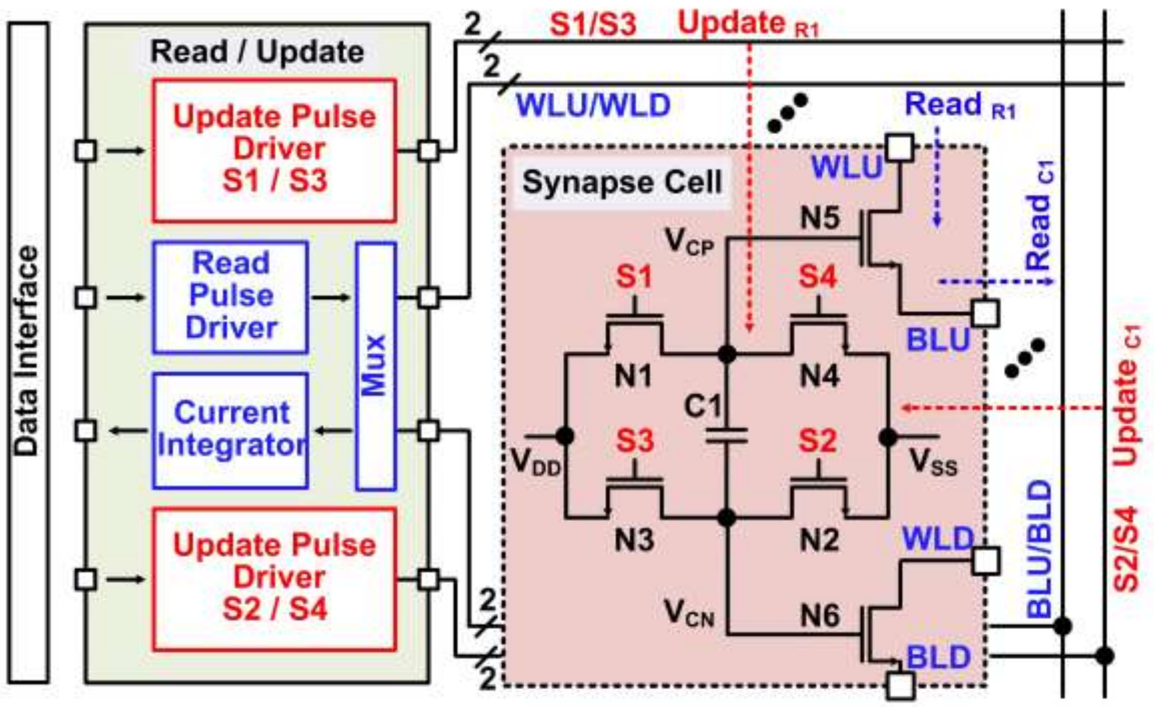

Figure S3. Operation diagram of the neuromorphic system with emphasis on the read and update functions with 6T1C synapse array.

The operation of the PCB for measuring the synaptic device is composed of a read mode that reads the conductance of the device and an update mode for weight update. The data flow in read mode is as follows. First, when output for each row is sent from the MCU, the corresponding output is simultaneously sent to the pulse driver through the data interface composed of D Flip Flop (DFF). The pulse driver is composed of Multiplexer (MUX) and transmits a voltage corresponding to a digital signal from the DC power supply to the WLU or WLD in Figure S3. To enable the backpropagation process, there is a MUX that connects WLU and BLU or WLD and BLD in reverse, and N5 and N6 are selected and read as another MUX. The conductance of the synaptic element reads the accumulated current through the ADC built into the MCU. Since the ADC of MCU operates at 0~3.3V, the value of $V(WLU) - V(WLD)$ is read by giving a negative value. Therefore, the direction of current flows from the output of the AMP to the pulse driver through the source and drains of N5, and N6. After the ADC sampling is finished, the current integrator is initialized through the switch. The update mode must be output more simultaneously than the read mode. Like the read mode, it controls sending a signal through the DFF at the same time and adjusts the voltage through the update pulse driver configured like the read pulse driver. First, it receives the signal that distinguishes potentiation and depression and decides whether to apply voltage to S1 and S2 or S3 and S4. Then, a voltage is applied to each transistor gate by operating the DFF clock according to the length of the input pulse. During potentiation, a voltage is applied to the S3 and S4 so that N3 and N4 do not operate. In addition, during the depression, a voltage is applied to the S1 and S2 so that N1 and N2 do not operate.

Discrete devices used in the PCB are as follows: MUX used NXP's 74hc4053, AMP and DFF were Ti's OP07x and SN74HC574, a switch was Vishay's DG201, and MCU used Atmel's sam3x8e based on arm cortex m3. Since the voltage operating range of the MUX is -5~5V, the PCB must be operated within the range. To remove noise as much as possible, the power supply voltage has $3300\mu F$, $1\mu F$, and $1nF$ capacitors, and each node has $1\mu F$, and $1nF$ capacitor. In addition, the used MCU has a 12-bit resolution, and the effective number of bits (ENOB) is 10.5-bit. The switching speed of the MUX takes at least 40ns and has a resistance of up to 140 ohms. 420ohms can be added to the synaptic resistance by going through at least three MUX, but it doesn't have much of an impact as it applies almost equally to all synapses.

S4. Comparison between rTT and TTv1[6] using an algorithm flow chart

TTv1[6]	r-TT algorithm
1 : initialize A = A_{ref} by measuring symmetric points and storing them into a separate reference matrix(A_{ref})<br>2: initialize C to random values<br>3: k=0<br>4 : t=0<br>5 : nr = Number of rows in A/C<br>6: ns = Hyperparameter − Number of update cycles<br>7: For each data in the training dataset	1 : A=A_{ref} (No need to set up a separate reference matrix. Reference point is automatically set to be leakage converging point which can be easily measured.)<br>2: initialize C to random values<br>3: k=0<br>4 : t=0<br>5 : nr = Number of rows in A/C<br>6: ns = Hyperparameter − Number of update cycles<br>7: For each data in the training dataset
8: $y = (\gamma(A - A_{ref}) + (C - C_{ref}))x$	8: $y = (C - C_{ref})x$
9: : $z = \left(\gamma\left(A - A_{ref}\right) + \left(C - C_{ref}\right)\right)^{T} \delta$	9: : $z = (C - C_{ref})^{T}\delta$
10: $a_{ij} \leftarrow a_{ij} + \eta_a[\delta_i \times x_j]F_{ij}(a_{ij})$- $\eta_a\|[\delta_i \times x_j]\|G_{ij}(a_{ij})$	10: $a_{ij} \leftarrow a_{ij} + \eta_a[\delta_i \times x_j]F_{ij}(a_{ij})$- $\eta_a\|[\delta_i \times x_j]\|G_{ij}(a_{ij})$<br>11 : $a_{ij} \leftarrow a_{ij} \times e^{-\frac{t}{RC}}$ // t: time taken from updating the core devices of one row to updating the core devices of the next row.
11: k=mode(k+1,ns)<br>12: If (k=0)	12: k=mode(k+1,ns)<br>13: If (k=0)
13: u=prepare_vector(t)	14: u=prepare_vector(t)
If device charateristics changed after cycling, a reference matrix(A_{ref}) needs to be reprogrammed by refining the symmetric points, which can be time-consuming.	If needed, the reference point can be remeasured quickly.

14: $v = (A - A_{ref})u$	15: $v_{N5} = (A_{N5} - A_{ref,N5})u$<br>16: $v_{N6} = (A_{N5} - A_{ref,N6})u$<br>17: $v = v_{N5} - v_{N6}$
15: $c_{ij} \leftarrow c_{ij} + \eta_c[f(v_i) \times u_j]F_{ij}(c_{ij})$-$\eta_c\|[f(v_i) \times u_j]\|G_{ij}(c_{ij})$	18: $c_{ij} \leftarrow c_{ij} + \eta_c[f(v_i) \times u_j]F_{ij}(c_{ij})$-$\eta_c\|[f(v_i) \times u_j]\|G_{ij}(c_{ij})$
16: t= mod(t+1, nr)<br>17: end<br>18: end	19: t= mod(t+1, nr)<br>20: end<br>21: end

Table S3. Comparison between rTT and TTv1 using algorithm pseudo-code.

S5. Conventional SGD learning results

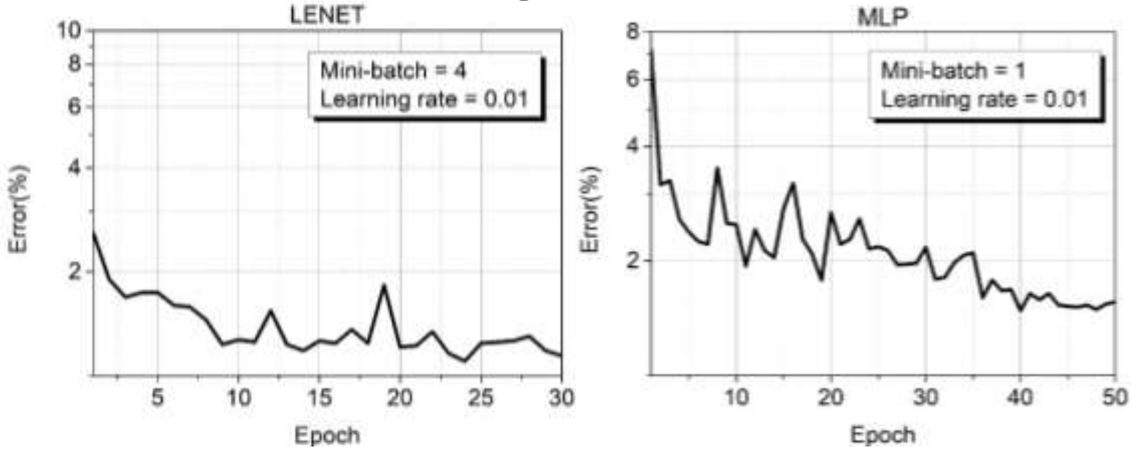

Figure S4. Conventional SGD learning results reflecting the characteristics of 6T1C devices in LENET5 and MLP structures. The average accuracy of the last 5 epochs of LENET5 and MLP is 98.744% and 98.484%, respectively.

S6. Extraction of NL values through linear regression analysis

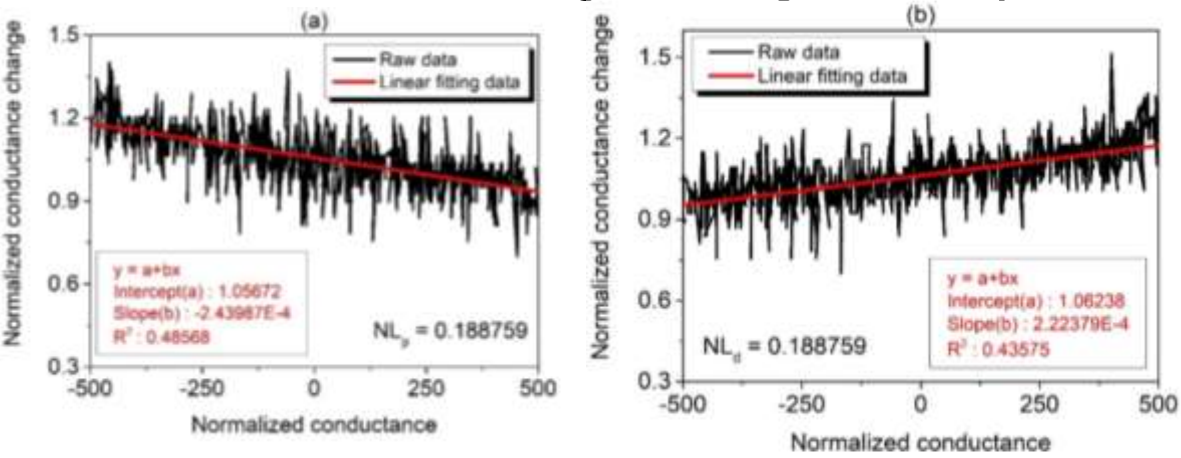

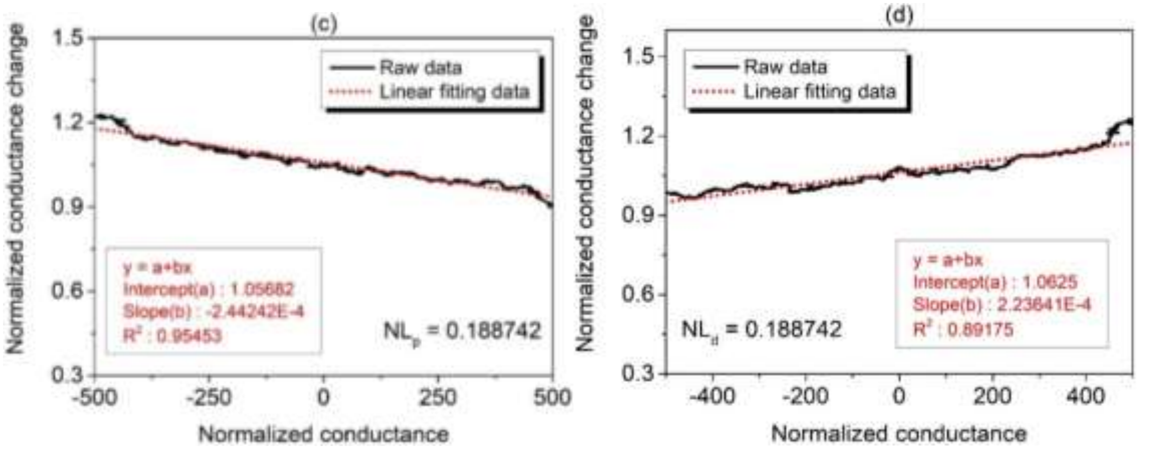

Figure S5. Result of extracting NL through linear regression analysis for NL ≈ 0.2 cases of main Figure 4a. In the case of (a) and (b), the R^2 value was low because the data was noisy. Therefore, NL was extracted by applying a moving average of 40 sections, and R^2 rose to ~0.9, but NL hardly changed. Therefore, it was confirmed that the device could extract the NL ≈ 0.2 level.

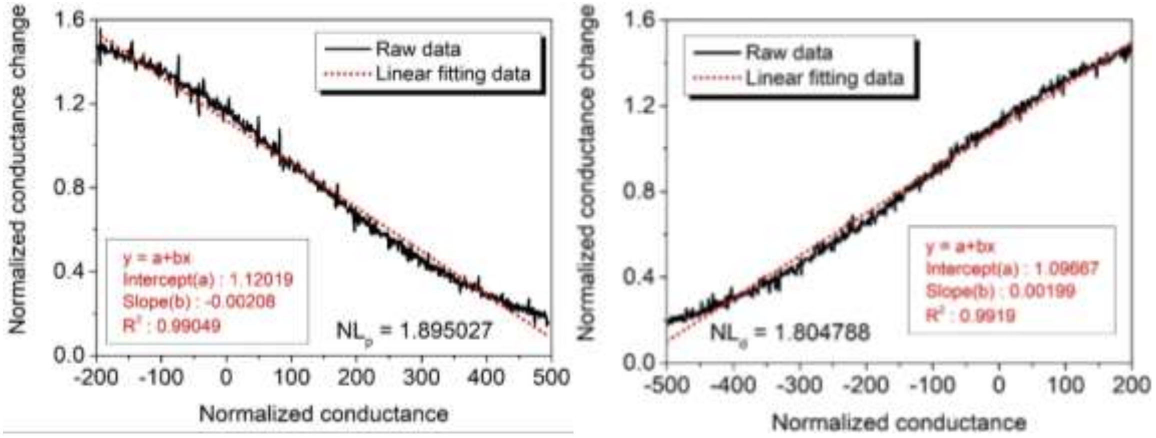

Figure S6. Result of extracting NL through linear regression analysis for NL=2.0 case main Figure.4a. During learning, max$|w_{ij}|$ is known as $1/\sqrt{n}$ where n is the number of layer input neurons.[7] Therefore, linear regression analysis was performed by extracting only the range of weights mainly used for learning. For potentiation, weight -0.4~1(normalized conductance -200~500), and for depression, weight -1~0.4(normalized conductance -500~200) range was used.

S7. TTv1, rTT learning result using 6T1C device

NL \ t/RC	5e-2	5e-3	5e-4	5e-5	5e-6	5e-7	5e-8	0
0.2	71.034	86.202	93.434	96.294	96.456	96.288	96.542	96.392
0.5	71.712	86.07	94.788	96.854	97.276	97	97.13	96.928

1.0	71.21	88.214	95.342	97.156	97.23	97.142	97.45	97.712
1.5	69.238	89.436	95.66	97.762	98.134	98.028	98.016	98.148
2.0	68.092	87.97	94.794	97.552	98.094	97.778	97.946	97.684

Table S4. TTv1 learning results according to various retention levels and NL of auxiliary device(6T1C). The neural network structure is LENET5, and the values in the table are the average learning accuracy of the last 5 epochs.

t/RC NL	5e-3	5e-4	5e-5	5e-6	5e-7	5e-8	0
0.2	82.06	89.758	94.386	96.888	96.634	96.682	96.634
0.5	81.316	90.176	94.64	96.708	96.554	96.814	96.886
1.0	81.302	89.596	94.77	96.924	96.81	97.116	97.256
1.5	81.984	90.48	94.982	97.196	97.206	97.312	97.21
2.0	80.968	90.344	95	97.19	97.426	97.516	97.584

Table S5. TTv1 learning results according to various retention levels and NL of auxiliary device(6T1C). The neural network structure is MLP, and the values in the table are the average learning accuracy of the last 5 epochs.

t/RC NL	5e-2	5e-3	5e-4	5e-5	5e-6	5e-7	5e-8	0
0.2	93.97	97.576	97.95	96.954	95.638	96.048	95.652	94.996
0.5	93.47	97.356	97.282	95.596	95.414	95.524	94.838	94.834
1.0	93.15	96.788	95.84	94.104	93.558	94.276	93.008	93.362
1.5	92.84	95.38	95.258	92.958	92.654	91.566	92.036	91.86
2.0	92.128	94.202	92.038	90.622	91.66	89.622	90.008	89.942

Table S6. rTT learning results according to various retention levels and NL of auxiliary device(6T1C). The neural network is LENET5, and the values in the table are the average learning accuracy of the last 5 epochs.

t/RC NL	5e-3	5e-4	5e-5	5e-6	5e-7	5e-8	0
0.2	93.122	96.736	96.598	96.256	96.018	95.932	96.026
0.5	91.882	97.008	97.09	96.574	96.368	96.398	96.546
1,0	93.122	96.736	96.598	96.256	96.018	95.932	96.026
1.5	93.568	96.3	96.112	95.784	95.368	95.466	95.04
2.0	94.116	95.454	95.588	94.904	94.732	95.07	94.858

Table S7. rTT learning results according to various retention levels and NL of auxiliary device(6T1C). The neural network is MLP, and the values in the table are the average learning accuracy of the last 5 epochs.

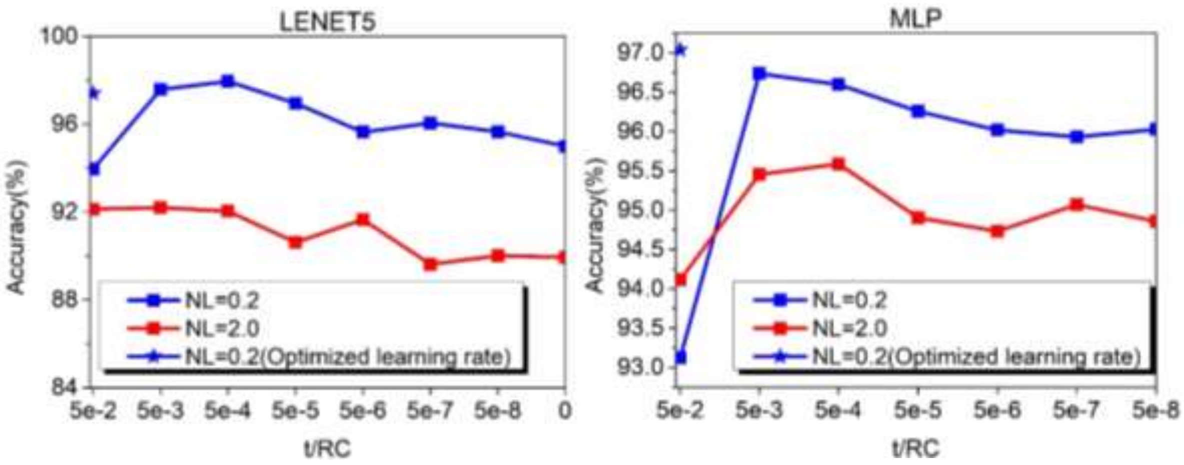

Figure S8. Neural training results by applying the rTT to NL=0.2, 2.0. The asterisk mark in the graph is the learning result applying the optimized learning rate. Therefore, if the optimal learning rate is applied for each learning situation, it can be demonstrated that learning is possible without deterioration in learning accuracy even in a wide retention range.

S8. 6T1C device using IGZO TFT and capacitor with extremely low leakage current

It is known that the capacitor with the lowest leakage current has a leakage current of ~0.25×10^{-24}A per 1fF at room temperature.[8] Therefore, a 10fF capacitor will have a leakage current of about ~0.25×10^{-23}A. Furthermore, the lowest leakage current is known to be less than 10^{-25}A per $1\mu m$ channel width at room temperature and the IGZO TFT fabricated with a channel width of 60nm has a leakage current of $\sim10^{-26}$A.[5] Therefore, when comparing the capacitor current level with the lowest leakage current and the IGZO TFT with the lowest leakage current level, the capacitor leakage current is ~400 times larger than that of the TFT, so the capacitor leakage current is dominant. If we calculate the RC time constant of the 6T1C device of 10fF capacitance/cell considering capacitor leakage, which is the dominant leakage path in the synaptic cell, it is as ~ $(1\times10^{-14}F)\times(4\times10^{23}\Omega)=4\times10^{9}s$. Therefore, the IGZO-based 6T1C device has a large very retention margin, so it is a device capable of applying complex neural network or large input datasets even with a 10fF capacitance/cell level device.

Reference

[1] S. Kim, T. Gokmen, H. M. Lee, W. E. Haensch, *Midwest Symp. Circuits Syst.* **2017**, *2017-Augus*, 422.

[2] D. Saito, J. Doevenspeck, S. Cosemans, H. Oh, M. Perumkunnil, I. A. Papistas, A. Belmonte, N. Rassoul, R. Delhougne, G. Kar, P. Debacker, A. Mallik, D. Verkest, M. H. Na, *IEEE Trans. Electron Devices* **2020**, *67*, 4616.

[3] S. Park, S. Seong, G. Jeon, W. Ji, K. Noh, S. Kim, Y. Chung, **2022**, *2200554*, DOI 10.1002/aelm.202200554.

[4] X. Duan, K. Huang, J. Feng, J. Niu, H. Qin, S. Yin, G. Jiao, D. Leonelli, X. Zhao, W. Jing, Z. Wang, Q. Chen, X. Chuai, C. Lu, W. Wang, G. Yang, D. Geng, L. Li, M. Liu, *Tech. Dig. - Int. Electron Devices Meet. IEDM* **2021**, *2021-Decem*, 10.5.1.

[5] T. Onuki, T. Atsumi, R. Tokumaru, T. Murakawa, K. Kato, C. M. Lai, D. Chen, S. Yamazaki, **2020**, *01*, 569.

[6] T. Gokmen, *Front. Artif. Intell.* **2021**, *4*, 1.

[7] M. J. Rasch, T. Gokmen, W. Haensch, (Preprint) arXiv: 1906.02698, submitted Jun **2019**, 1.

[8] H. Baba, S. Ohshita, T. Hamada, Y. Ando, R. Hodo, T. Ono, T. Hirose, Y. Kurokawa, T. Murakawa, H. Kunitake, T. Nakura, M. Kobayashi, H. Yoshida, M. C. Chen, M. H. Liao, S. Z. Chang, S. Yamazaki, *Tech. Dig. - Int. Electron Devices Meet. IEDM* **2021**, *2021-Decem*, 21.2.1.
